# Formononetin isolated from *Sophorae flavescentis* inhibits B cell-IgE production by regulating ER-stress transcription factor XBP-1

**DOI:** 10.3389/falgy.2022.1056203

**Published:** 2023-02-01

**Authors:** Nan Yang, Ibrahim Musa, Anish R. Maskey, Ke Li, Zhenzhen Wang, Banghao Liang, Shuwei Zhang, Jixun Zhan, Xiu-Min Li

**Affiliations:** ^1^ General Nutraceutical Technology LLC, Elmsford, NY, United States; ^2^ Department of Pathology, Microbiology & Immunology, New York Medical College, Valhalla, NY, United States; ^3^ Guangdong Hospital Department of Integrated Traditional Chinese and Western Medicine, Guangzhou University of Chinese Medicine, Foshan, China; ^4^ Department of Chinese Medicine, Henan University of Chinese Medicine, Zhengzhou, China; ^5^ Department of Biological Engineering, Utah State University, Logan, UT, United States; ^6^ Department of Otolaryngology, New York Medical College, Valhalla, NY, United States

**Keywords:** asthma, antiasthma simplified herbal medicine intervention (ASHMI), formononetin, IgE inhibition, IgE heavy chain, XBP-1

## Abstract

**Rationale:**

IgE plays an important pathologic role in most, if not all, allergic conditions. We previously showed that ASHMI (anti-asthma herbal medicine intervention) suppressed IgE production in murine models of asthma and in asthma subjects. However, the active compounds in ASHMI responsible for the IgE suppression are still unknown.

**Objective:**

We sought to identify the compound(s) in ASHMI that are responsible for IgE inhibition as well as investigate the mechanisms by which the identified compound(s) decreases IgE production.

**Methods:**

The compounds in *Sophorae Flavescentis* were separated using Column chromatography and preparative-HPLC. The separated compounds were identified using LC-MS and ^1^H-NMR. U266 cells, an IgE-producing plasma cell line, were cultured with various concentrations of identified compounds. The levels of IgE production by the U266 cell were measured by ELISA. Trypan blue exclusion was used to determine the cell viability. The gene expression of XBP-1 and IgE-heavy chain was determined by RT-PCR.

**Results:**

A single compound identified as formononetin was isolated from *Sophorae Flavescentis*. Formononetin significantly and dose dependently decreased the IgE production in U266 cells across a concentration range of 2–20 µg/ml (*p* < 0.05–0.001 vs. untreated cells) with an IC50 value of 3.43 μg/ml. There was no cytotoxicity at any tested concentration. Formononetin significantly decreased XBP-1, and IgE-heavy chain gene expression compared with untreated cells (*p* < 0.001).

**Conclusion:**

Formononetin decreased IgE production in human B cell line U266 cells in a dose-dependent fashion through the regulation of XBP-1 ER transcription. Formononetin may be a potential therapy for allergic asthma and other IgE-mediated diseases.

## Introduction

There has been a steady increase in the prevalence of allergic diseases around the world. Allergy is now the 6th leading cause of chronic illnesses in the United States, with more than 50 million people suffering from the disease yearly (1). Globally about 300 million people suffer from asthma while about 30 million have allergies to some type of food (2). Most allergic diseases are IgE mediated. IgE is the central molecule that has been studied to be responsible for degranulating mast cells and basophils. It is one of the immunoglobulin isotypes, with the lowest abundance in the serum of normal individuals at a minute concentration of 50–200 ng/ml (3). Concentration of IgE in allergic individuals has been shown to increase to 10-fold that of normal serum levels (4). In order to trigger an allergic reaction, the ingested or inhaled allergen will bind to IgE which is bound to the FcεRI high affinity IgE receptors present on the surface of mast cells or basophils. This causes the crosslinking of the receptors and downstream degranulation to release the pharmacologic mediators responsible for clinical symptoms seen in allergic reactions (5).

The therapies approved for use in treatment of allergic diseases or that are currently undergoing clinical trials mostly function by binding circulating IgE; they do not directly suppress the production of IgE from plasma cells. Omalizumab (6), Ligelizumab (7), and MEDI4212 (8) are monoclonal antibodies that have been approved or are undergoing clinical trials, they function by neutralizing serum IgE. Quilizumab (9), Bsc-IgE/CD3 (10), and XmAb7195 (11) are monoclonal antibodies that have undergone clinical trials to treat IgE-mediated diseases by targeting IgE^+^ B cells leading to the decrease in the number of these cells, which, in turn, leads to a decrease in the production of serum IgE. Suplatast tosilate inhibits the production of IL-4 and IL-5 in Th2 cells, which causes a decrease in the synthesis of IgE, It has been used in the treatment of allergic asthma (12–16). Most of the current therapies used in treatment of IgE function by binding to circulating IgE, depleting IgE^+^ B cells or suppressing the production of Th2 cytokines. However, they are expensive, are associated with adverse reactions and do not offer long term protection.

ASHMI, Antiasthma Simplified Herbal Medicine Intervention, is a non-steroidal Chinese herbal formula with components; *Ganoderma lucidum (GL)*, *Sophorae flavescens (SF)*, and *Glycyrrhiza uralensis (GU)*. ASHMI is effective in the treatment of Allergic Asthma, with clinical trials demonstrating acceptable levels of safety and tolerability (17, 18). Previous studies on chronic asthma murine model showed that ASHMI significantly reduced the Th2 cytokine levels and the OVA specific IgE levels vs. Sham group (19, 20) with *sophorae flavescentis* and *ganoderma lucidum* identified as the main components of ASHMI responsible for the Th2 cytokine suppression (21, 22). Compounds isoliquiritigenin, 7,4’ dihydroxyflavone (7, 4’-DHF), and liquiritigenin, isolated from *g. uralensis* were identified as the active compounds that are responsible for the suppression of the Th2 cytokines IL-4 and IL-5. Among these compounds, 7, 4’-DHF showed the highest potency on Th2 cytokine suppression (23). However, IgE inhibitory compounds from ASHMI remained to be identified. In this study, we sought to isolate and identify the bioactive components from ASHMI formula, which has IgE inhibitory effect *in vitro*, and investigate the mechanisms behind it.

## Material and methods

### Material and herbal products

RPMI 1640 and penicillin-streptomycin were purchased from Mediatech Inc (Manassas, VA), Fetal Bovine Serum (FBS) was purchased from Atlanta Biologicals (Lawrenceville, GA). 2-Mercaptoethanol (2-ME), sodium pyruvate, [3-(4,5-dimethylthiazol-2-yl)-2,5-diphenyltetrazolium bromide] (MTT) and dimethyl sulfoxide (DMSO) were purchased from Sigma Aldrich (St Louis, MO). Acetonitrile, dichloromethane, ethylacetate, methanol, and formic acid were purchased from Fisher Scientific (Pittsburgh, PA).

ASHMI product is a dried aqueous extract of SF, GU, and GL, in capsule form (17). ASHMI and the water extract of all three herbal medicines were manufactured in a GMP-certified facility, the Sino-Lion Pharmaceutical Company in Weifang, China (17). All the herbs used for manufacture originated from China. The raw herbs used meet acceptable quality criteria as defined by the Pharmacopoeia of People's Republic of China (24). Briefly, the raw herbs were cut into small pieces and soaked in water for 60 min, then boiled for 2 h. The procedure was repeated twice, and the decoctions were collected and concentrated under vacuum (18).

### Fractionation of radix *Sophora flavescens* and identification of active compounds

Dichloromethane extract of SF was performed utilizing liquid-liquid extraction method. Briefly, 50 g of *SF* provided by Sino-lion Pharmaceutical Company (P.R. China), was dissolved into 4 L water, and mixed with 3 L of CH_2_Cl_2_ in liquid-liquid extractor. 24 h later, the non-polar fraction was collected and dried on a rotary evaporator under low pressure. Methanol (300 ml) was used to re-dissolve the dried residues. The mixture was centrifuged at 2,500 rpm for 30 min and further separated with an AutoPurification system coupled with UV/Vis Detector (Waters, MA). Four fractions were collected based on the major peaks presented in the analytic HPLC chromatogram data.

### Analysis of licorice flavonoids by liquid chromatography-mass spectrometry (LC-MS) and nuclear magnetic resonance spectroscopy (NMR)

Compounds characterization was done using a Waters 2695 HPLC system (Waters Corporation, Milford, MA) coupled to a Waters Micromass LCT Premier mass spectrometer. Samples were separated on an analytical Column: Zorbax® SB C18 (5.0 µm, 4.6 × 150 mm) (Agilent Technologies, Santa Clara, CA) with 0.1% formic acid as mobile phase A and acetonitrile with 0.1% formic acid as mobile phase B. A gradient program was used as follows: 0–75 min, linear change from A–B (98:2, v/v) to A–B (52:48, v/v). A 15 min equilibration time was used before each HPLC run. The flow rate was set at 1 ml/min. The UV spectra were recorded between 200 and 460 nm. The detection wavelength was set at 254 nm, as previously described (25).

Molecular weight was identified using a Time-of-Flight mass spectrometer (LC-MS/TOF, Micromass LCT Premier, Waters Corporation) in both electrospray (ESI) positive and negative mode. 10 μl of sample was analyzed under the same gradient condition described above. The parameters were set as: Capillary Voltage: 3,200 v; Cone Voltage: 15 v; Aperture I: 25 v; Desolvation Temperature: 300°C; Source Temperature: 110°C; Desolvation gas: 500 L/h; Nebulizing gas: 40 L/h; Ionization mode: Electrospray; Positive Ion Acquisition Range: m/z 50–1,000. The results were collected and analyzed by Empower and Masslynx software (25).

^1^H NMR spectra were obtained on a JOEL instrument at 300 MHz using DMSO-*d_6_* as the solvent (25).

### IgE producing human U266 myeloma and cell culture

The human U266 myeloma cell line, purchased from ATCC (American Type Culture Collection; Rockville, MD) is widely used in the allergy research to screen the effects of compounds on B cell IgE production (26). Cells were cultured at 37°C under 5% CO_2_ in complete media containing RPMI 1640 medium supplemented, 10% FBS, 1 mM sodium pyruvate, 1 × 10^−5^ M β-ME and 0.5% penicillin-streptomycin. Cells were grown at an initial concentration of 2 × 10^5^ cells/ml. The cells were treated with different concentrations of ASHMI, individual herb extracts, and purified compounds after 6 days culture. Supernatants were harvested, and IgE levels were determined by using an ELISA Kit (Mabtech Inc, OH). In some experiments, U266 cells were treated with active compound for 3 days. Cells were then subjected to western or qPCR analyses.

### IgG producing ARH-77 cell culture

ARH-77 cell line, purchased from ATCC (American Type Culture Collection; Rockville, MD) to screen the effects of compound formononetin on B cell IgG production (27). Cells were cultured at 37°C under 5% CO_2_ in complete media containing RPMI 1640 medium supplemented, 10% FBS, 1 mM sodium pyruvate, 1 × 10^−5^ M β-ME and 0.5% penicillin-streptomycin. Cells were grown at an initial concentration of 2 × 10^5^ cells/ml. The cell culture was incubated with test compound at concentration of 20, 10, 5, 2.5 (μg/ml) on day 0, supernatants were harvested after 3 days culture, and IgG levels were determined by using an ELISA Kit (Mabtech Inc, OH).

### Human U266 and ARH-77 cell viability *via* trypan blue exclusion

Cell viability was evaluated using a trypan blue exclusion as previously described. Briefly, a 10 µl of cells suspension from each culture was mixed with equal volume of trypan blue dye (23). The mixture was loaded into a hemocytometer and cells were counted under a microscope. The percentage of viable cells was calculated as follows: Viable cells (%) = (total number of viable cells)/(total number of cells) × 100.

### CCK-8 assay

Cell cytotoxicity was evaluated using cell counting kit-8 according to manufacturer's instruction, and previously published including ours (28, 29). We cultured U266 cells as described 2.4 and incubated with test compound formononetin at concentrations of 20, 10, 5 and 2.5 (μg/ml) for 3 days in a 96 well plate. 10 μl of CCK-8 was added to each well, after 10 min of incubation at 37°C, the optical density was read using a microplate reader at 450 nm.

### Real time polymerase chain reaction (RT-PCR)

Quantitative real time PCR was used to detect the expression of Xbp-1 and (IgE-H). U266 (1.5 × 10^6^ cells/ml) were co-incubated with formononetin (10 µg/ml) at 37°C under 5% CO_2_ for 3 days. Cells were then harvested, and total RNA was isolated using Trizol reagent (Gibco BRL, Rockville, MD) following the manufacturer's instructions. The RNA concentrations were then quantified by triplicate optical density (OD) readings (Bio-Rad SmartSpect 3000; Bio-Rad, Hercules, CA). The reverse transcription was performed to get the cDNA using RevertAid RT Reverse Transcription Kit (ThermoFisher, Waltham, MA) as described by the manufacturer's instructions. Pre-chill sterile, thin-walled tubes and reaction tubes on ice, pre-chill nuclease-free water. Combine the RNA (up to 1 µg) and the cDNA primer in nuclease-free water for a final volume of 5 µl per RT reaction on ice. Place the tube into preheated 70°C heat block for 5 min immediately chill the tube on ice for 5 min spin each tube to collect all condensate. Prepare the reverse transcription reaction mix ImProm-II™ 5X Reaction Buffer, MgCl_2_, dNTP, Recombinant RNasin® Ribonuclease Inhibitor, ImProm-II™ Reverse Transcriptase with nuclease-Free Water to a final volume of 15 μl. Add 15 µl of revese transcription reaction mix to each reaction tube containing the 5 µl RNA and primer mix. Final volume is 20 µl. Use PCR to finish the RT reaction at 25°C for 5 min, 42°C for 60 min, 70°C for 15 min. The RT-PCR amplification was performed using Maxima™ SYBR Green qPCR Master Mix (2X) kit (ThermoFisher, Waltham, MA) with epsilon germline primers or GAPDH primers. Primers were synthesized by Sigma-Aldrich Corporation (St. Louis, MO), see Supplementary Table S1 for the primer list.

### Western blotting

We used western blotting analysis to measure the protein U266 cells at 3.0 × 10^6^ cells/ml were cultured with formononetin at a concentration of 10 (µg/ml) for 3 days. After the culture time point the cells were harvested, centrifuged at 3,000 rpm for 5 min and the supernatant discarded. The cell suspension was washed with PBS and centrifuged at 3,000 rpm for 5 min. Then the cell lysis was done for protein extraction using 100–200 µl of RIPA lysis buffer, with intermittent vigorous vortexing and incubating on ice for about 1 h. After completion of lysis, the tubes were spined in centrifuge at 14,000 rpm for 20 min at 4 degrees Celsius. The supernatant was collected and transferred to another tube to measure the protein concentration. Electrophoresis was done at 90 V for 15 min followed by 100–110 V for the remaining time and transferred to a PVDF membrane. The membrane was incubated with primary antibodies of the XBP-1, β-actin overnight. The next day, the membrane was washed with PBST and incubated with secondary antibody for 2 h and after the membrane was washed with PBST 4 times before adding the chemiluminescence substrate and imaging. The primary antibodies of XBP-1 and β-actin were ordered from Cell Signaling (Danvers, MA, USA) and were used at a 1:1,000 dilution, the secondary antibody was used at a 1:10,000 dilution.

### Statistics

All statistical analyses were performed using Sigma Stat 3.5 (Systat Software Inc., Chicago, IL) or GraphPad Prism (version 9, GraphPad Software, Inc, San Diego, CA). One way ANOVA (analysis of variance) was performed followed by Bonferroni correction for all pairwise comparisons. For skewed data, the differences between the groups were performed by one way ANOVA on rank followed by Donne's method for all pairwise comparisons. *p*-values were determined by a two-sided calculation. *p* value ≤ 0.05 was considered as statistically significant.

## Results

### ASHMI formula and its herbal constituents inhibited IgE production in a dose dependent manner

**Figure 1:**
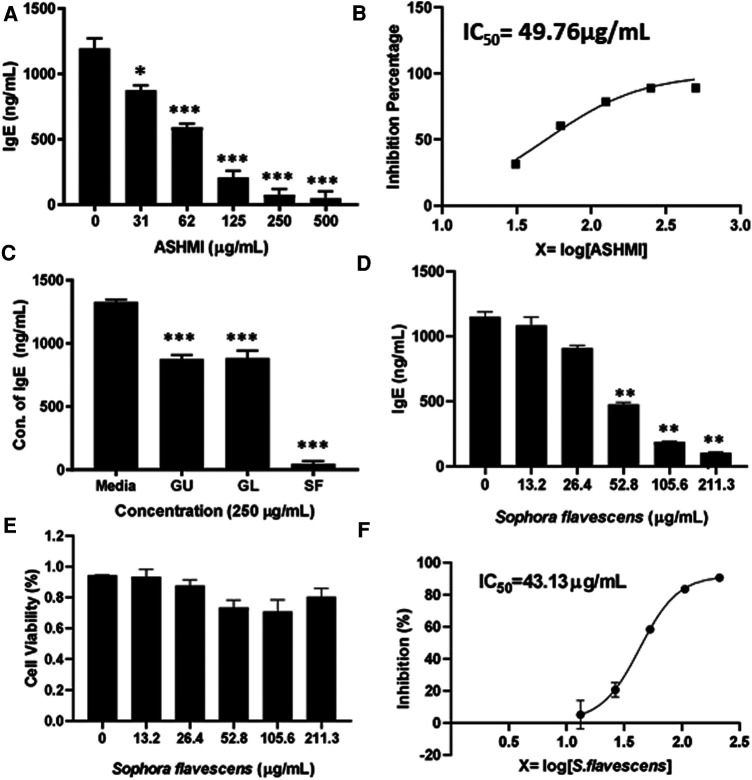
Dose-dependent effect of ASHMI formula and its herbal constituents on IgE production in U266 cells. (**A**) Inhibitory effect of ASHMI formula on IgE production by U266 cells. (**B**) Percentage of IgE inhibition vs. log [ASHMI concentration] curve. (**C**) Inhibitory effect of individual herbal constituents of ASHMI on IgE production at 250 µg/ml. (**D**) Inhibitory effect of *Sophora flavescens* on IgE production *in vitro*. (**E**) Cell viability of *Sophora flavescens* on U266 cells. (**F**) Percentage of IgE inhibition vs. log [*Sophora flavescens* concentration] curve. U266 cells (2 × 10^5^ cells/ml) were cultured with ASHMI and individual herbal constituents at concentrations as indicated for 6 days. The supematants were harvested and IgE levels were measured by ELISA. Results were expressed as the mean ± SD. *p < 0.05; **p < 0.01; ***p < 0.001. Data represents triplicate experiments.

To evaluate the inhibitory effect of ASHMI formula on IgE production, human myeloma cell line U266 cells were cultured with ASHMI at different concentrations (0, 31, 62, 125, 250 and 500 µg/ml) for 6 days. After the culture time point, the cells were harvested, supernatant was collected and using ELISA IgE concentration was measured. Results showed that ASHMI formula significantly inhibited IgE production in a dose-dependent manner (Figure 1A, *p* < 0.05; *p* < 0.001) with an IC_50_ value of 49.76 µg/ml (Figure 1B). Trypan blue exclusion analysis showed that ASHMI did not affect cell viability at the concentrations tested (Figure 1C). *Sophora favescens*, *Glycyrrhiza uralensis*, and *Ganoderma lucidum* (three herb components of ASHMI) were cultured with U266 cells at a concentration of 250 µg/ml each. All three herbs significantly inhibited the production of IgE in U266 cells. Of the three herbs *Sophora flavescens* had the highest inhibitory effect on IgE production when compared to the others *Glycyrrhiza uralensis* and *Ganoderma lucidum*. The IgE inhibition percentage of *Sophora flavescens* is 96.54% ± 3.01%, while *Glycyrrhiza uralensis* and *Ganoderma lucidum* were 33.90% ± 5.63% and 33.35% ± 8.20% respectively (Figure 1C, *p* < 0.001). Further experiment showed that *Sophora flavescens* dose-dependently inhibited IgE production (Figure 1D, *p* < 0.01) with an IC_50_ values of 43.13 µg/ml (Figure 1F) without any significant toxicity (Figure 1E).

### *Sophora flavescens* dichloromethane extract and its sub fractions inhibit IgE production in U266 cells

**Figure 2:**
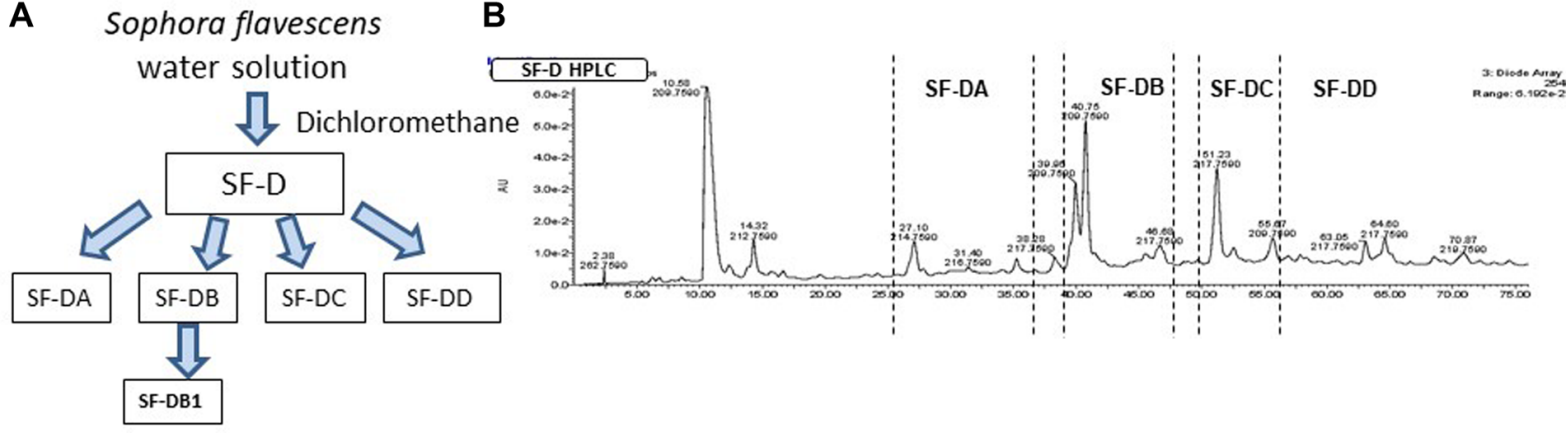
Compound isolation process of *Sophora flavescens*. (**A**) Flow chart of isolation and purification process of compounds from *Sophora flavescens*. (**B**) HPLC chromatogram of further separation using Auto-Purification system.

**Figure 3:**
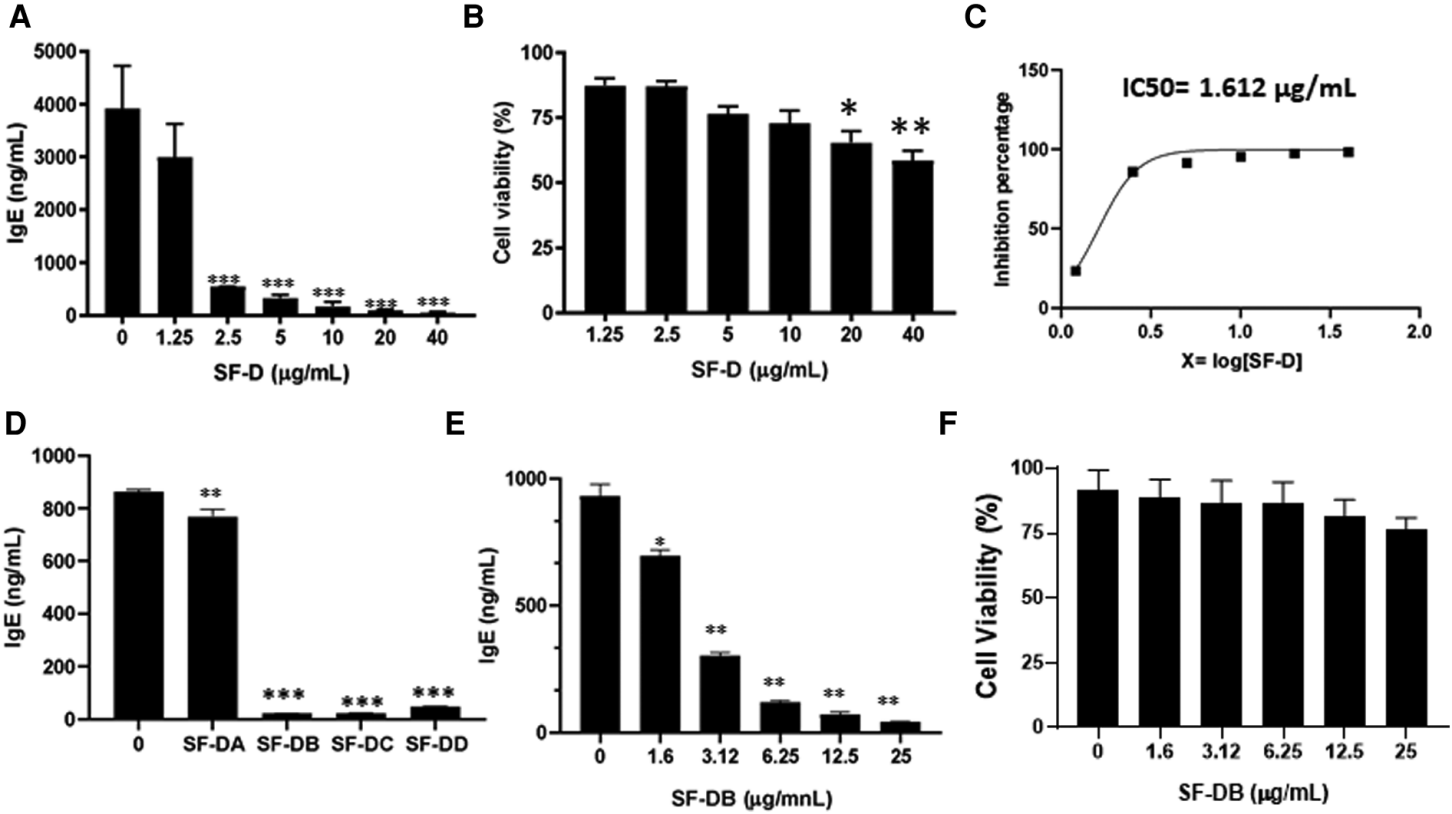
Different fractions of *Sophora flavescens* on IgE production in U266 cells. (**A**) Inhibitory effect of SF-D fractions on IgE production by U266 cells. (**B**) Cell viability of cultured U266 cells with SF-D fractions. (**C**) Percentage of IgE inhibition vs. log [SF-D concentration] curve. (**D**) Inhibitory effect of SF-D-sub-fractions, SF-DA, SF-DB, SF-DC, SF-DD on IgE production by U266 cells. (**E**) Dose-dependent experiment of SF-DB on IgE production by U266 cells. (**F**) Cell viability of cultured U266 cells with or without SF-DB.*p < 0.05; **p < 0.01; ***p < 0.001. Data represents triplicate experiments.

The process of isolating the active compounds in *Sophora flavescens* is shown in (Figure 2A). Organic solvent dichloromethane was used to isolate extract from *Sophora flavescens*. The extraction was collected and named SF-D. SF-D was further separated using Waters Auto-Purification system. Four subfractions were collected using auto-purification system based on the polarity (Figure 2B). Active compounds were isolated through a bioactivity guided fractionation process. The dichloromethane extract of *Sophora flavescens*, SF-D, was tested for its inhibitory effect on IgE production. The result showed that SF-D inhibits IgE production in a dose dependent fashion (Figure 3A, *p* < 0.001) with an IC_50_ value of 1.612 µg/ml (Figure 3C) without significant cytotoxicity (Figure 3B). The four sub-fractions of SF-D were also tested for their inhibitory effect on IgE. Results showed that all four subfractions significantly inhibited IgE production, with an inhibition percentage of; SF-DA −10.93% ± 3.17%; SF-DB −97.37% ± 0.03%; SF-DC −97.30% ± 0.25%; and SF-DD −94.37% ± 0.1% (Figure 3D, *p* < 0.01; *p* < 0.001). We next focused on SF-DB for further study because this fraction contains two major peaks, in addition to its potency of IgE suppression. SF-DC fraction showed one major peak and several small peaks and had very low yield therefore it would be difficult to isolate the pure compound from SF-DC. SF-DD was the last collection of the fraction process (i.e., it was collected as the washout) that contains many small peaks; therefore, it would be difficult to isolate pure compounds. Prior to further isolation and purification of SF-DB compounds, we tested SF-DB dose dependent effect on IgE production. It was shown that SF-DB dose-dependently inhibited the IgE production in a non-toxicity manner (Figure 3E, *p* < 0.05; Figure 3F, *p* < 0.01, Figure 3F). Further separation process was performed using auto purification system and one compound was purified. The purity of this compound was >96% as determined by analytical HPLC.

### Highly potent sub-fraction of *Sophora flavescens* identified as formononetin

**Figure 4:**
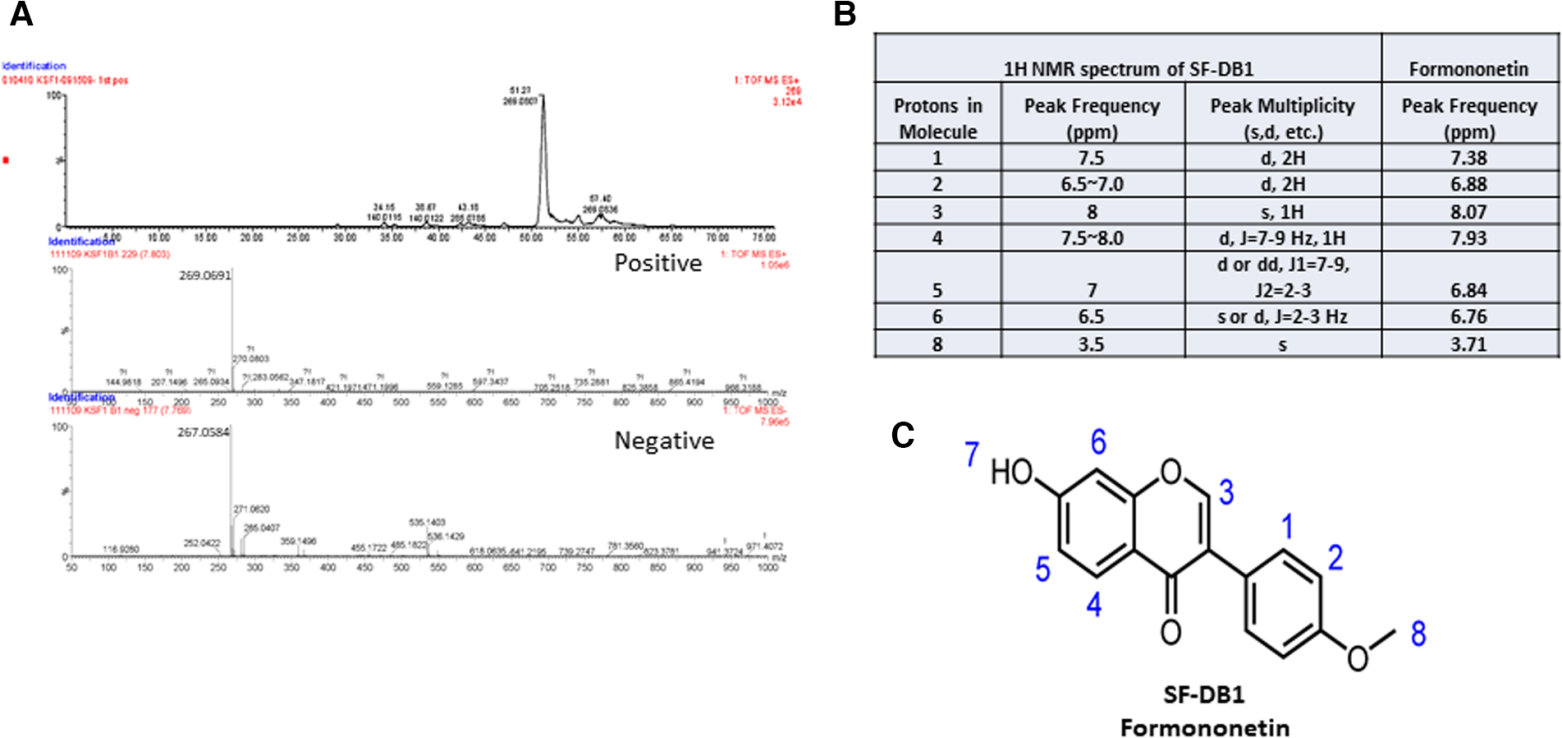
Characterization of isolated compound. (**A**) Mass spectra of SF-DB1 in both positive and negative mode. (**B**) ^1^H NMR spectra data of SF-DB1 (Formononetin). (**C**) Chemical structures of isolated compound, Formononetin.

The molecular weight of the major compound isolated from fraction SF-DB was determined with LC-MS in both positive and negative mode (Figure 4A). Mass spectra data showed a significant [M + H]^+^ ion peak at m/z 269 and [M-H]^−^ value of 267, and ^1^H-NMR spectrum was shown in Supplementary Figure S1) The molecular weight (MW) of this compound was then determined to be 268 g/mol. The ^1^H NMR data defined the isolated compound is formononetin (Figure 4B); this is consistent with previously reported data of formononetin (30). Thus, based on the LC-MS and ^1^H-NMR data, this isolated compound was identified as formononetin (Figure 4C). In addition, we also isolated a second compound and identified as maackiain by ^1^H-NMR (Supplementary Figure S2). However, the isolation efficiency of maackiain is very low using our prep HPLC system. We, therefore focused on formononetin for further anti IgE effect and underlying mechanisms.

### Formononetin inhibits IgE production in U266 cells without affecting IgG in ARH-77 cells

**Figure 5:**
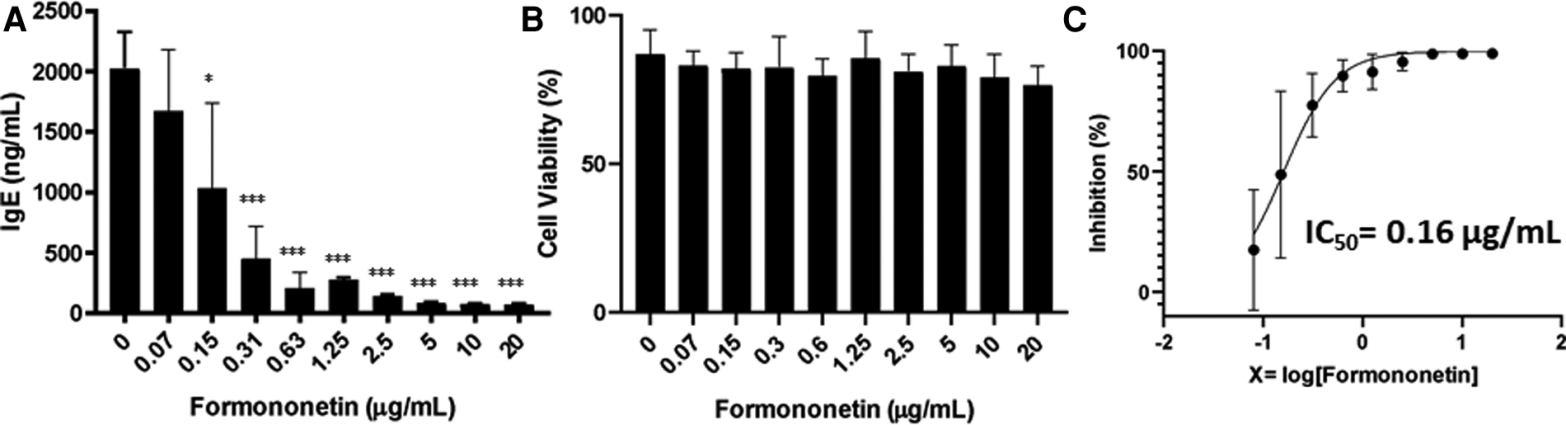
Formononetin inhibits IgE production in U266 cells in a dose dependent manner. (**A**) Inhibitory effect of formononetin on IgE production by U266 cells. (**B**) Cell viability of cultured U266 cells with or without formononetin. (**C**) Percentage of IgE inhibition vs. log [formononetin concentration] curve. *p < 0.05; **p < 0.01; ***p < 0.001. Data represents triplicate experiments.

To further examine the bioactivity of formononetin on IgE production, U266 cells were co-cultured with formononetin at concentrations (0.15, 0.31, 0.63, 1.25, 2.5, 5, 10, 20 µg/ml). The results showed that formononetin dose dependently inhibited the IgE production by U266 cells (Figure 5A, *p* < 0.05; *p* < 0.001). Formononetin significantly inhibited the IgE production *in vitro* at the concentration as low as 0.15 µg/ml. No significant toxicity was observed at concentrations lower than 1.25 µg/ml (Figure 5B). The IC_50_ value of formononetin was calculated as 0.16 µg/ml (Figure 5C). Having identified the compound formononetin, we obtained formononetin commercially from Sigma Aldrich, (St. Louis, MO), and tested at different concentrations (2.5, 5, 10, 20 µg/ml), using ELISA, we measured IgE, the results showed that formononetin dose dependently inhibited IgE production in U266 (Supplementary Figure S3A, *p* < 0.001). No significant cytotoxicity was observed as indicated by trypan blue measure (Supplementary Figure S3B) and cytotoxic measure using CCK-8 assay (Supplementary Figure S3C). To investigate if the inhibitory mechanism of formononetin is specific to IgE we cultured commercial formononetin with ARH-77 cells and measured the IgG production. Our result showed that formononetin did not significantly change the IgG production in the ARH-77 cells (Supplementary Figure S4).

### Formononetin inhibits the XBP-1 and IgE heavy chain mRNA expression in U266 cells

**Figure 6:**
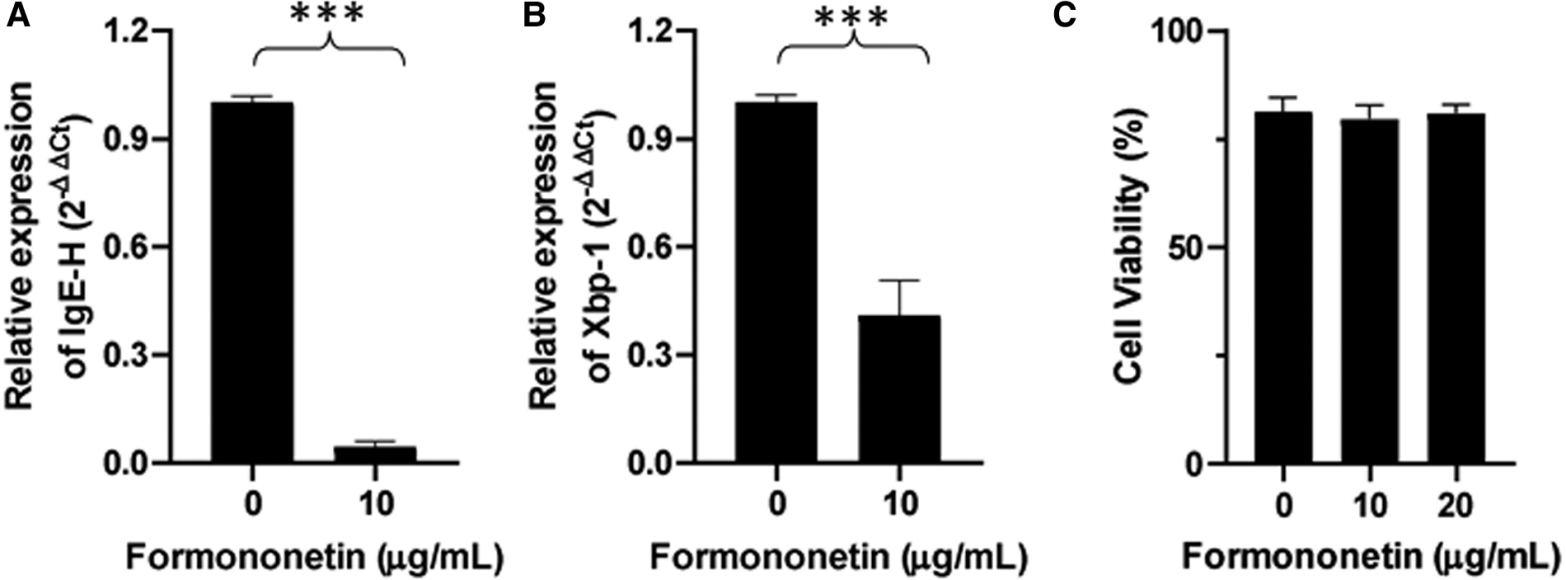
Formononetin inhibits the XBP-1 and IgE heavy chain mRNA expression in U266 cells. The relative expression level of IgE heavy chain (**A**) and XBP-1 (**B**) were determined by comparing with GAPDH mRNA expression. Fold change were calculated by the 2(−ΔΔCt) method. (**C**) Cell viability of formononetin on U266 cells at 10 and 20 µg/ml. Data are shown as the mean ± SEM. ***p < 0.001. Data represents triplicate experiments.

IgE heavy chain transcription is a critical step for IgE isotype switching. To investigate the mechanism by which formononetin inhibits IgE production, mRNA expression of IgE heavy chain and XBP-1 was measured using real-time PCR. Human U266 cells were cultured with formononetin at 10 µg/ml, the total RNA in U266 cells was extracted and real time-PCR experiment was performed. Results showed that both the relative mRNA expression of IgE Heavy chain and XBP-1 were significantly downregulated by formononetin (Figure 6, *p* < 0.001) when compared to the untreated culture group.

### Formononetin inhibits the XBP-1 protein expression in U266 cells

**Figure 7:**
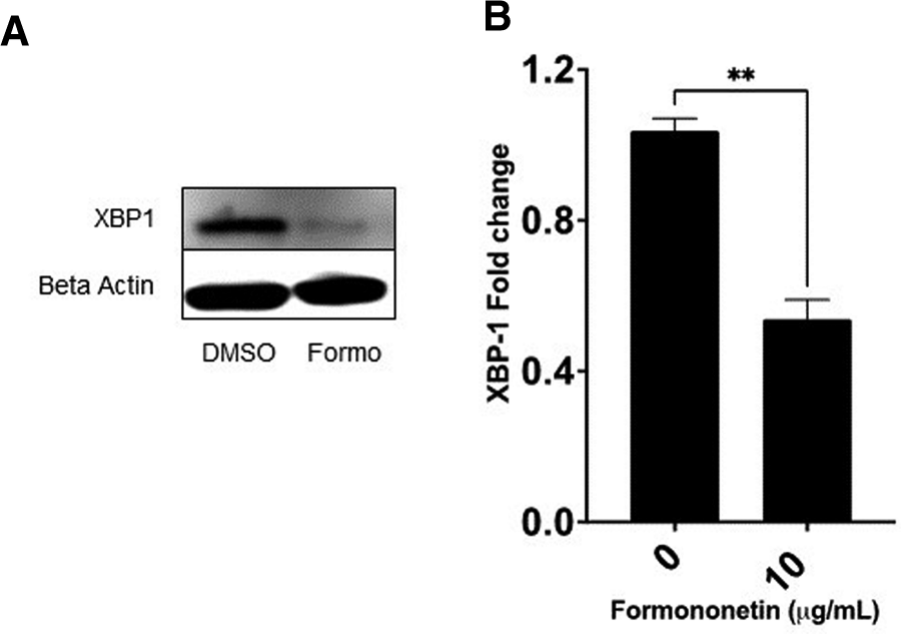
Formononetin inhibits protein XBP-1 protein expression. U266 cells were cultured with formononetin for 72 h and protein expression was determined using western blotting. (**A**) Western Bands (**B**) XBP-1 protein expression. Data represents triplicate experiments and expressed as mean ± SD. **p < 0.01 vs. control (0.1% DMSO).

The gene expression of XBP-1 was significantly decreased by formononetin, therefore we used western blotting to measure the effect of formononetin on the protein expression of XBP-1. Our results showed that formononetin significantly inhibited the protein expression of XBP-1 (Figure 7, *p* < 0.01).

## Discussion

One of the herbal constituents of ASHMI, *Sophora flavescens*, was shown to be effective in reducing IgE. *Sephora flavescens* is an herb clinically used for the treatment of viral hepatitis, viral myocarditis, enteritis, gastrointestinal dysfunction, and skin diseases such as colpitis, psoriasis and eczema. SF was previously determined to have anti-inflammatory properties, interfering specifically with the TNF-α cytokine, which accounts for its traditional use in inflammatory-related disease (31). It was found that the main bioactive components of the herb are major alkaloids found to have antipyretic properties (32). However, alkaloids alone do not work to combat inflammatory response related to type I hypersensitivity and allergy related responses instigated through IgE. Therefore, it became important to investigate essential isoflavanoids in order to determine possible IgE inhibitory compounds. Further analysis using preparative HPLC showed that a subfraction of *sephora flavescens* was very effective in reducing IgE, and this fraction was identified using ^1^H-NMR analysis to be formononetin. Formononetin is a compound that belongs to a class of naturally occurring active compounds known as flavonoids. Flavonoids are an important class of natural products that have favorable biochemical and antioxidant, anti-inflammatory, anti-mutagenic and anti-carcinogenic effect associated with diseases such as cancer, alzheimer, atherosclerosis, and allergic diseases. Several studies have shown that flavonoids decrease IgE production. Therefore, it became important to investigate essential isoflavonoids in order to determine possible IgE inhibitory compounds. Formononetin isolated from *Sephora flavescens* one of the three main components of ASHMI is a compound that belongs to a class of naturally occurring flavonoid. Therefore, we sought to investigate the mechanism by which formononetin decreases IgE production using human B cell line U266.

Formononetin at a concentration of 20 µg/ml significantly decreased IgE production compared to zero treatment on U266 cells. This data is consistent with previous results obtained from different studies in which the whole herb extract was seen to decrease serum IgE level in mice. Our data suggested that formononetin is the main active compound responsible for IgE inhibition in SF. Cell viability results also showed that the formononetin has a high safety profile at the dose dependent concentration tested on U266 cells. Further study needs to be done to investigate the ability of formononetin to decrease IgE production in the PBMCs of patients with allergies.

IgE production requires class switch recombination between large and highly repetitive switch (S) regions. The human ɛ-germline gene promoter region consists of several promoters which includes STAT6 promoter region and NF-ĸB region. NF-ĸB binds to two sites in the ɛ-germline gene promoter and cooperates with STAT6 in synergistic action of transcription (39). The activity of STAT6 is highly influenced by NF-ĸB (33, 34). At the conclusion of Ig class switching, a B cell secretes one of the different classes of antibodies (35). Each of the three stages of class switching—germ line gene transcription, DNA recombination and B cell differentiation is very complex resulting in the activation of ɛ-germline gene transcription is essential (36).

Upon activation mature B cells undergo immunoglobulin class switch recombination and differentiate into antibody-secreting plasma cells (37). Antagonistic influences from two groups of transcription factors control the differentiation of B cells into plasma cells (38). The group of transcription factors that maintain B cell includes Pax5, Bach 2, and Bcl6 while the group of transcription factors that promote and facilitate plasma cell differentiation include Irf4, Blimp1 and XBP-1 (37). XBP-1 is a positively acting transcription factor in the CREB/ATF family that is expressed at a high level in plasma cells (37). XBP-1 acts downstream of Blimp-1 gene to regulate a broad complement of genes encoding ER-associated proteins, many of which are involved in protein secretion (38). XBP-1 induces physical expansion of the ER, in addition to increasing cell size, organelle biosynthesis, total protein synthesis. This shows that XBP-1 plays a critical role in the secretion of immunglobulins (38). XBP-1 is uniformly expressed in all multiple myeloma cell lines (38) (U266 cell is a multiple myeloma cell line). Because of the role of XBP-1 in secretion of immunoglobulin we decided to investigate if whether it is inhibited in human B cell line U266 cultured with formononetin. U266 cells were cultured with 20 µg/ml of formononetin, and the cells were harvested after 6 days for RNA isolation and RT-PCR was done to analyze gene expression levels. Our result showed that formononetin decreased the XBP-1 gene expression significantly when compared with untreated cell cultures. We believe that XBP-1 inhibition is another mechanism that formononetin uses in decreasing IgE secretion. This is the only study to show that formononetin inhibits XBP-1 in human B cell line U266 cells.

Activation of ɛ germline gene is an essential step towards IgE expression (35, 36), which leads to generation of ɛ mRNA transcripts after class switch recombination. The presence of IgEH transcripts in the B cell provides evidence that class switch recombination has occurred in the germline region. In our experiment, we used an IgE-producing myeloma cell line. This cell line already underwent class switch recombination to IgE. As such, the expression of IgE heavy chain in these cells is largely under control of the promoters located 5’ of the variable (V) gene and the enhancers located between the joining and constant genes in the IgH. Therefore, we used RT-PCR to compare the relative expression of IgE heavy chain transcripts in the U266 cultured with formononetin against untreated cultures. The result showed that there was a significant decrease in IgEH mRNA transcripts in the treated culture compared to untreated cultures. This finding indicates that formononetin may be a potential therapeutic in established IgE-mediated allergies such as food allergy. Studies are underway to investigate formononetin effect in peanut allergic murine model. Nevertheless, the limitation of this study include that we were unable to determine the effect of formononetin on IgE class switch recombination (CSR) using this cell line. Further studies are needed using a primary B cell line to investigate if indeed formononetin alters germline transcription and possibly prevents class switch recombination.

## Conclusion

We, for the first time, demonstrated that *Sophora flavescens*, one of the three herbal constituents of ASHMI, is the major component which inhibited the IgE production. Formononetin, isolated from *Sophora flavescens*, significantly inhibited IgE production *in vitro* without altering cell viability. The IgE inhibitory mechanisms of formononetin might be through the regulation of XBP-1.

## Data Availability

The original contributions presented in the study are included in the article/**Supplementary Material**, further inquiries can be directed to the corresponding author.
