# Supplementary figures and images for: Formononetin isolated from Sophorae flavescentis inhibits B cell-IgE production by regulating ER-stress transcription factor XBP-1
Source: Front Allergy. 2023 Feb 1;3:1056203. doi: 10.3389/falgy.2022.1056203 (PMC9928687; doi:10.3389/falgy.2022.1056203)

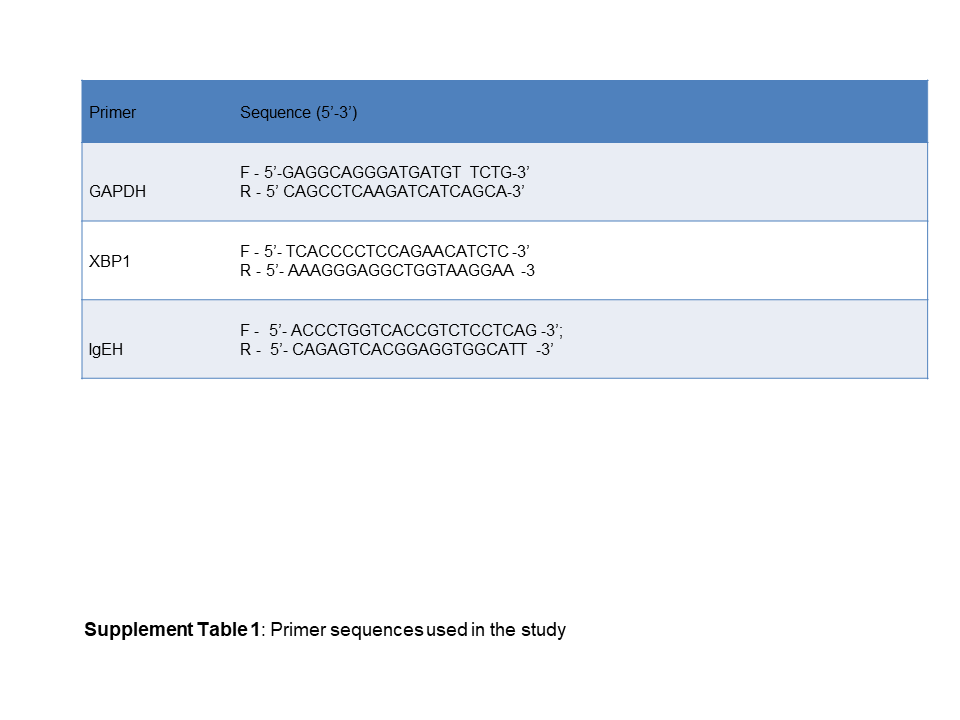

Supplement: Supplementary file 1 [file Image1.tif]

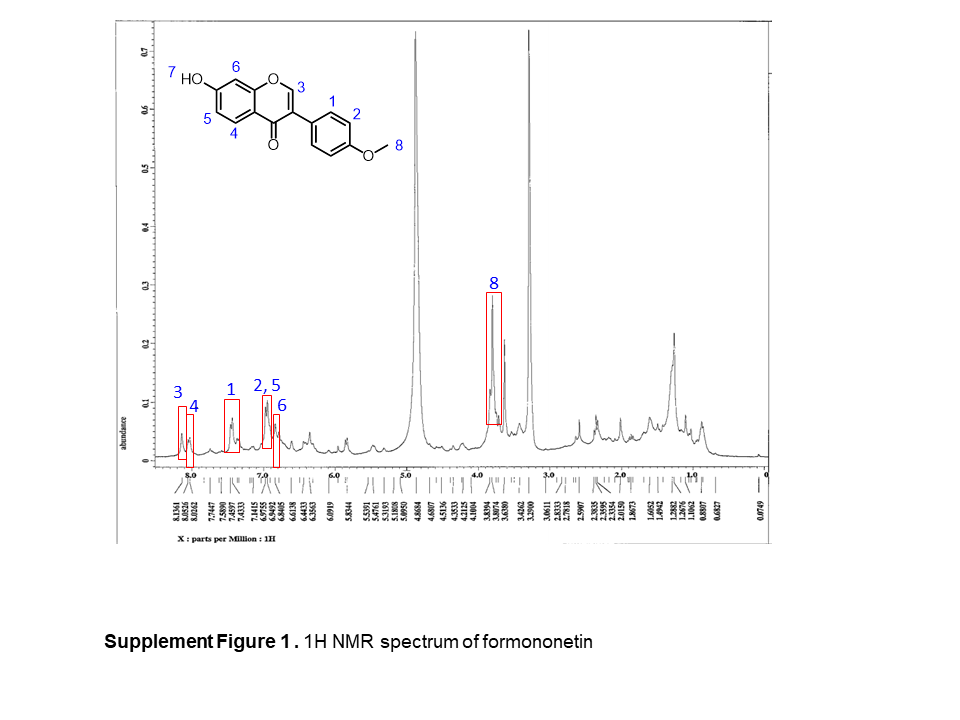

Supplement: Supplementary file 2 [file Image2.tif]

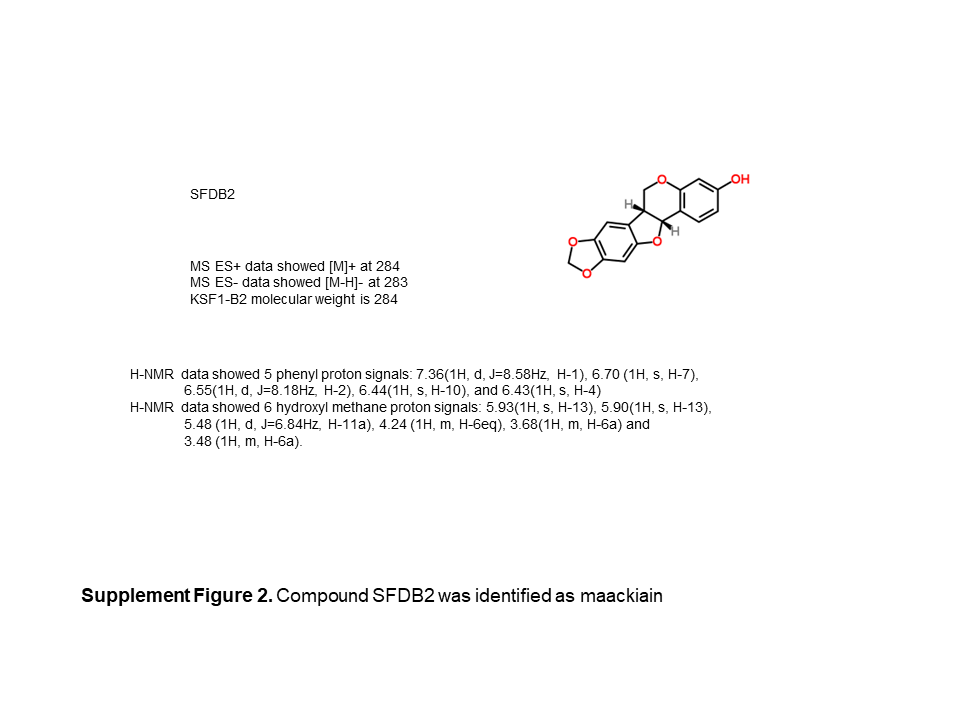

Supplement: Supplementary file 3 [file Image3.tif]

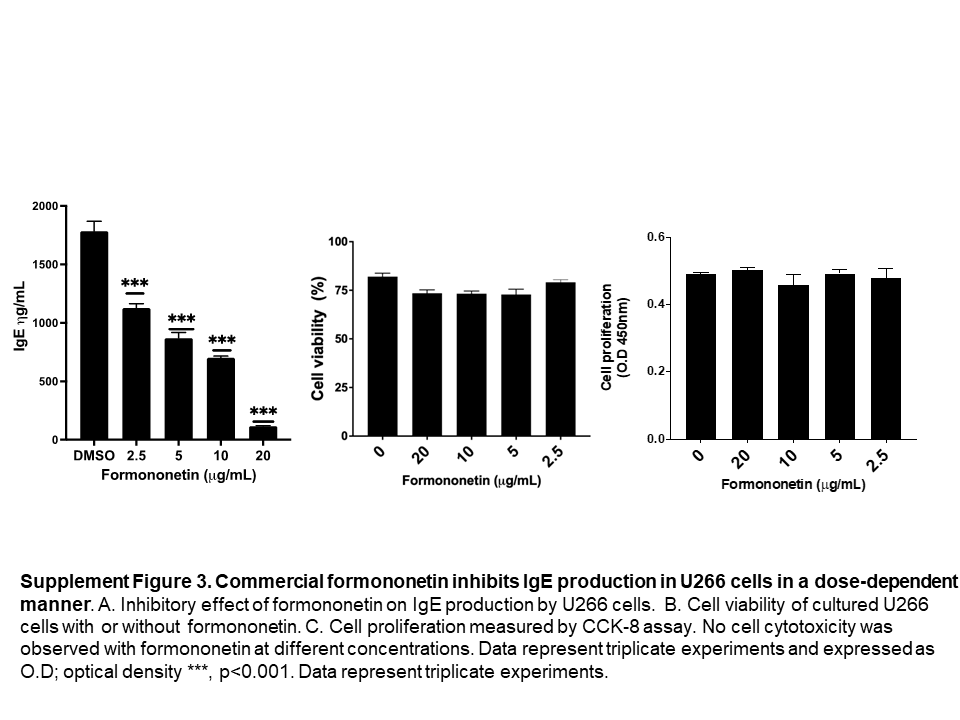

Supplement: Supplementary file 4 [file Image4.tif]

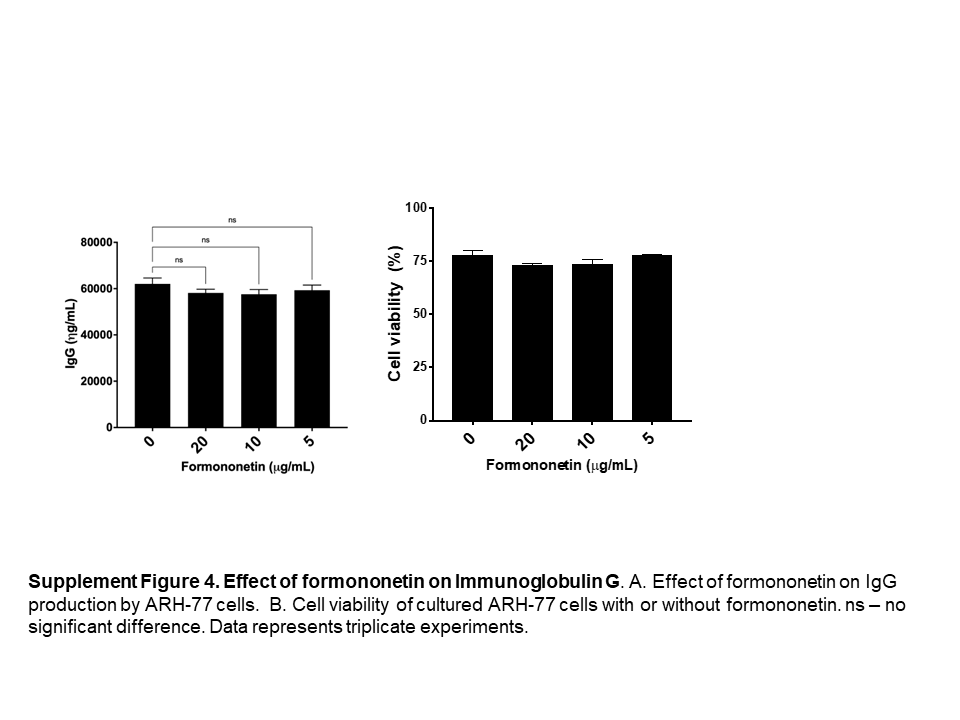

Supplement: Supplementary file 5 [file Image5.tif]
